# PEG-Fusion Repair After Peripheral Nerve Injuries Enhances Behavioral Recovery and Reduces Self-Mutilation in Rat Models

**DOI:** 10.3390/neurolint18050083

**Published:** 2026-04-28

**Authors:** Liwen Zhou, Cathy Z. Yang, George D. Bittner

**Affiliations:** Department of Neuroscience, The University of Texas at Austin, Austin, TX 78712, USA

**Keywords:** autophagia, sciatic functional index, Von Frey, neuropathic pain, sciatic nerve injuries, polyethylene glycol

## Abstract

**Background/Objectives:** Self-mutilation behavior is often triggered by neuropathic pain associated with peripheral nerve injuries (PNIs). Polyethylene glycol (PEG)-fusion is a repair method that rapidly joins/fuses the open ends of closely apposed severed axons, greatly reduces Wallerian degeneration, and restores sensorimotor behavior much more rapidly than current clinical procedures. Here, we examined whether the improved sensorimotor behavior recovery following PEG-fusion repair of sciatic nerve injuries compared to Negative Controls (NC) correlated with self-mutilation. We also examined six variables (repair method, behavioral tests, sex, injury type, strain, and surgical experience) that could influence self-mutilation outcomes. **Methods:** The Sciatic Functional Index (SFI) and the Von Frey (VF) behavioral tests were performed and analyzed. Regression and other analyses were performed to determine the independent effect of six variables on self-mutilation rates and severity. **Results:** PEG-fused rats that had no self-mutilation had significantly better SFI scores than those that had self-mutilation. More rapid VF sensory recovery in PEG-fused rats was also associated with less self-mutilation. Self-mutilation rates and severity were: (1) significantly reduced following PEG-fusion repairs compared to NCs; (2) significantly increased following weekly VF tests; (3) not different between female and male rats or (4) between simple transection and segmental-loss PNIs; (5) non-existent in Lewis rats and significantly less severe in Sprague Dawley rats than Long Evans rats; and (6) significantly reduced in rats operated on by experienced PEG-fusion surgeons who historically achieved better SFI outcomes than trainee surgeons. **Conclusions:** Our data suggest potential clinical benefits of PEG-fusion repair to produce more rapid and better sensorimotor recoveries and reductions of self-mutilation behaviors.

## 1. Introduction

Simple transection or segmental-loss peripheral nerve injuries (ST- or SL-PNIs) often result in immediate loss of sensorimotor functions [1,2,3,4]. Anucleate distal or autograft/allograft axonal segments undergo Wallerian degeneration within 1–3 days. Functional recovery depends on slow axon regeneration by outgrowth (1–2 mm/day) from surviving proximal axons connected to a somal nucleus. These axonal outgrowths may take weeks to months to reinnervate distal targets in rats and months to years in humans [5,6]. The current experimental and clinical standard treatment for ST-PNIs is neurorrhaphy, in which severed nerve stumps are closely apposed by micro-sutures through the epineurium to facilitate axonal regeneration from the proximal surviving axons [1,7]. For SL-PNIs that produce large gap defects, grafting materials are typically used in combination with neurorrhaphy to bridge the defect [1,3,6,7,8].

Polyethylene glycol (PEG)-fusion has recently emerged as a promising alternative strategy to repair ST- and SL-PNIs [9,10,11,12,13,14,15,16,17,18,19,20,21,22,23,24,25,26]. This technology combines neurorrhaphy with a well-specified sequence of bioengineered solutions, one being 3.35 kDa PEG, to non-selectively fuse open, closely apposed, viable axons. PEG-fusion repair produces: (1) immediate morphological and electrophysiological continuity across lesion sites, (2) prevention of Wallerian degeneration of many axons, (3) immediate re-innervation of distal sensory and motor targets, and (4) significantly better sensorimotor behavioral recovery within 6–8 weeks compared to neurorrhaphy-only Negative Controls (NCs). Additionally, PEG-fused donor viable peripheral nerve allografts (VPNAs) that are neither immune-suppressed nor tissue-matched used to repair SL-PNIs are not immunologically rejected—unlike conventional, non-PEG-fused VPNAs [9,10,11,12,13,14,15,16,17].

Peripheral nerve injuries not only produce significant sensorimotor impairments but also neuropathic pain, a complex behavior that has been documented both experimentally [27,28,29,30,31,32,33,34,35,36,37,38,39,40] and clinically [41,42,43,44,45,46,47,48]. Neuropathic pain symptoms include sensory deficits (allodynia, hyperalgesia, and spontaneous pain) and self-mutilation (autophagia) in animals and humans. Neuropathic pain might be influenced by factors such as nerve repair methods, injury types, sex and sex hormones, animal strains, environmental enrichments, animal handling and diet, etc. As described above, PEG-fusion enhances the recovery of sensorimotor functions in animal models in preclinical studies [9,10,11,12,13,14,15,16,17] and humans in case studies and clinical trials [49,50,51,52]. However, PEG-fusion effects on neuropathic pain and self-mutilation have not been previously studied.

In this study, we hypothesized that PEG-fusion would reduce self-mutilation behavior and enhance sensorimotor recovery assessed by Sciatic Functional Index (SFI) and Von Frey (VF) behavioral assays. We analyzed the rate and severity of self-mutilation in more than 700 rats that received sciatic ST- or SL-PNIs in previous or current PEG-fusion studies. We examined and statistically analyzed the effects of the following six variables: repair method (PEG-fusion vs. NC), sensory testing (VF or no VF), sex, injury type (ST- or SL-PNIs), strain (Sprague Dawley (SD), Long Evans (LE), or Lewis), and extent of surgical experience for PEG-fusion repair. Our results support our hypothesis, show several variables (repair methods, VF assays, strain, and surgical experience) that increase or decrease self-mutilation rate and severity, and are consistent with much other evidence that PEG-fusion is a safe and effective technology to repair PNIs.

## 2. Materials and Methods

### 2.1. Animals

All experimental procedures were conducted in accordance with the *National Institutes of Health Guide for the Care and Use of Laboratory Animals* [53] and were approved by the Institutional Animal Care and Use Committee at the University of Texas at Austin (AUP20252200278, approved 9 June 2025). Male (250–500 g) and female (225–300 g) outbred SD and LE and inbred Lewis rats aged 3–6 months were housed 2–3 per cage with a PVC tube enrichment under a 12 h light/dark cycle. Food and water were provided ad libitum. Operated rats also receive wood block as enrichment. All surgical and behavioral procedures were conducted during the day.

A total of 360 sciatic-operated rats (344 SD and 16 LE) were analyzed in different subsets for self-mutilation rates and severity. As controls, additional sets of sciatic-operated Lewis rats (*n* = 347), Unoperated Control SD rats (*n* = 20; 10 males and 10 females), and Sham-operated (*n* = 18; 9 males and 9 females) Control SD rats were tested weekly for SFI/VF assays and showed no self-mutilation at any time. All rats were sourced from the current study, previous studies [10,11], and ongoing related experiments. All surgeries, except for 19 SD rats operated on by trainee surgeons analyzed for surgical experience comparisons, were performed by three surgeons with comparable experience and procedural success (325 SD, 16 LE, and all controls).

### 2.2. Surgical Procedures

Surgical procedures were performed as previously described [9,10,11,14,15]. Rats were anesthetized with isoflurane (4% induction, 2% maintenance; [RXISO-250; Animal Health International, Roanoke, TX, USA]) in oxygen at 1.5 L/min (Handlebar Anesthesia, Pflugerville, TX, USA). The lateral aspect of the left hindlimb was shaved, sterilized, and incised (1–2 cm) through the skin and the biceps femoris to expose the sciatic nerve in a Ca^2+^-containing isotonic solution (0.9% NaCl with 2 mM CaCl_2_), which mimics physiological extracellular conditions. The sciatic nerve was left intact in Sham-operated Control rats, but exposed to all surgical solutions except PEG—as described below.

The sciatic nerve was either sharply transected or ablated (~5–10 mm segment) at mid-thigh using fine dissection scissors. Sciatic nerve stumps were then irrigated with the hypotonic antioxidant 0.5% methylene blue (prepared from 1% methylene blue; BioPharm, Iselin, NJ, USA) to open the axonal ends, reduce intracellular vesicle formation that seals plasmalemmal lesions, and enhance visualization of epineurium during surgery. A hypotonic (250 mOsm), calcium-free diluted Normosol-R solution (prepared from Normosol-R; [21300905; Animal Health International]) was applied as needed to maintain the axonal ends open and keep the tissue moist prior to PEG-fusion. For transection repair, the sciatic nerve stumps were closely apposed by at least four 10–0 micro-sutures through the epineurium (neurorrhaphy). For ablation repair, size- and sex-matched fresh donor VPNAs were trimmed to 1–3 mm longer than the gap and transplanted. Both host nerve stumps were trimmed and closely apposed to the graft by neurorrhaphy. For PEG-fused rats, neurorrhaphy was then followed by application of 50% *w*/*w* 3.35 kDa PEG (Sigma-Aldrich, St. Louis, MO, USA) in distilled water for 1–2 min. PEG-fusion dehydrates phospholipid membranes, thereby facilitating rapid and non-specific reconnections of closely apposed severed axons. NC rats received no PEG solution after neurorrhaphy. The rats were given unique IDs posted on cage cards and randomly assigned to PEG-fused or Negative Control groups to eliminate bias in surgical procedures.

Following neurorrhaphy (NC), PEG application (PEG-fused), or sham operation, all surgical sites were flushed several times with Ca^2+^-containing isotonic extracellular solution to seal any remaining holes in cell membranes of injured sciatic nerves. [NC rats receive all solutions except PEG.] The surgical site was closed with 5–0 sutures, and the skin was closed with wound clips. Rats were allowed to recover on heating pads before returning to standard housing. Carprofen (5 mg/kg, subcutaneous; [17613254; Animal Health International]) was administered during surgery and for three days post-operatively (PO). After operations, rats received PO monitoring and treatments approved in our IACUC protocol.

### 2.3. Sciatic Functional Index (SFI) Tests

SFI tests were performed and scored blinded as previously described [9,10,11]. Briefly, rats were handled and trained before surgery and tested weekly PO. Each rat with their blue inked left paw and red inked right paw was placed at one end of a slightly inclined board (1.52 m long, 10.2 cm wide) lined with paper strips and allowed to run to its home cage at the opposite end. SFI scores were calculated using the formula: (73*((NPL-EPL)/EPL+(ETS-NTS)/NTS+(EIT-NIT)/NIT)), where EPL and NPL stand for experimental and normal paw length, ETS and NTS for experimental and normal toe spread, and EIT or NIT for experimental and normal intermediate toe spread [54]. Unoperated rats typically exhibited SFI scores between −20 and +20, indicating gait symmetry. Impaired movement of the left-operated hindlimb resulted in negative SFI scores ranging from −90 to −120. Functional recovery was defined as previously published [12,16], e.g., achieving and maintaining an SFI score greater than −69 or −79 for ST- or SL-PNIs, respectively, until the endpoint that was often at least 6 weeks PO.

### 2.4. Dorsal Up–Down Von Frey Tests

VF tests were performed and scored blinded by two testers per session as previously described [11]. Rats were randomly assigned prior to surgery to either receive weekly VF tests or no VF tests to minimize selection bias. Briefly, rats were handled and trained before surgery and tested weekly PO. The starting hind paw (left-operated vs. right-intact) was randomly selected. The first tester gently restrained rats in towels and elevated them to expose the hind paws, while the second tester applied VF filaments (Stoelting Co., Wood Dale, IL, USA) when the rat was calm. The starting filament was #5.07 (10 g bending force). Based on the withdrawal response (positive or negative), the next filament’s force was decreased or increased, respectively. Five filament tests were conducted per hind paw, with at least 30 s of rest between filament tests. An adjustment factor of 0.5 was either added or subtracted from the threshold value of the last filament depending on whether the final response was negative or positive, respectively. VF threshold was converted to gram-force using the following equation: Force = 10^(0.904×filament #−3.54)^. Any response was voided and retried if the filament slipped or moved the hind paw or if the rat struggled in the towel during tests.

Rats that did not respond to the largest filament used (#6.10:100 g bending force) were considered unresponsive and assigned a VF threshold of 133.8 g. Yet-larger filaments (#6.45 (180 g) and above) were avoided because they did not elicit withdrawal responses at early PO times and caused long-lasting skin damage. The status of VF sensory recovery of the injured paw before self-mutilation onset was categorized as follows: “yes” if a rat showed any VF response before self-mutilation and “no” if no VF response occurred at any time before or the week right after self-mutilation.

### 2.5. Assessment of Self-Mutilation

Operated rats were included in this study based on the following criteria: **1.** Were intended to be maintained for at least 6 weeks PO (many do not reach 6 weeks PO due to early euthanasia); **2.** Received a sciatic ST-PNI or SL-PNI followed by PEG-fusion or NC repair; **3.** Exhibited no other complications that may affect SFI, VF, or self-mutilation behavior (e.g., tumor growth; *n* = 2). Rats that had sciatic nerve severance and additional nerve severances (e.g., sciatic and saphenous; *n* = 3 SD rats) were excluded because combined nerve injuries resulted in significantly worse self-mutilation than sciatic injury alone in some rat strains [29,55]. The same injuries to Lewis rats (*n* = 20) produced no self-mutilation.

Self-mutilation was monitored daily until the PO endpoint. PO care was coordinated among laboratory personnel using shared tracking logs that recorded animal ID, strain, sex, source, operation date, operation type, observations, self-mutilation date, and treatment schedule. To reduce bias, veterinarian staff were blind to the animal operation details and always confirmed and recorded the degree of injury following injury reports.

**Figure 1:**
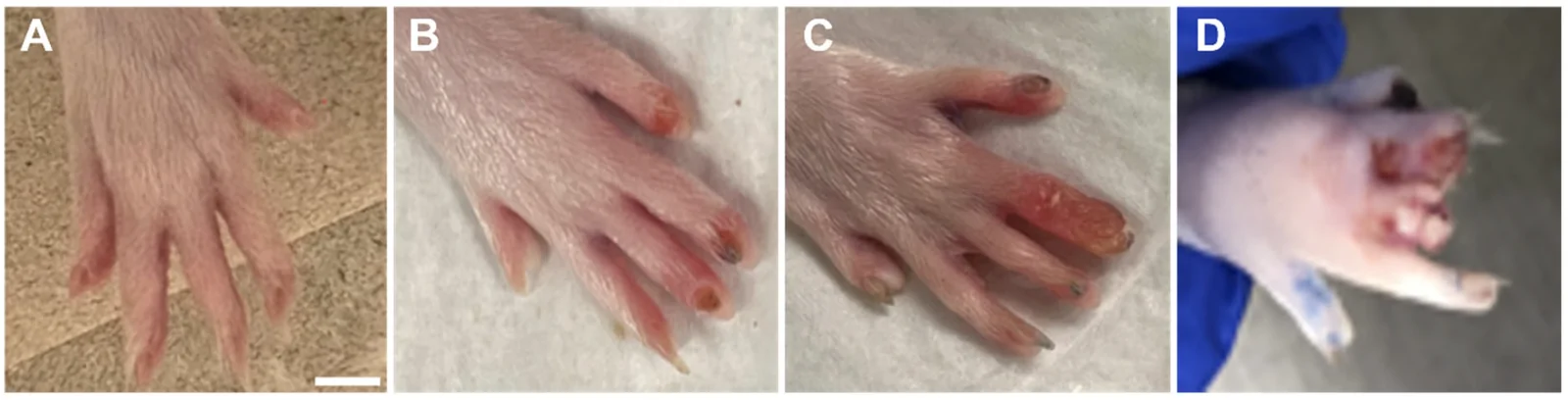
**Representative examples of different degrees of self-mutilation in Sprague Dawley rats following sciatic nerve injury and repair.** The hind paws on the operated side were monitored daily PO for signs of soft tissue damage. (**A**) No visible damage. (**B**) Mild self-mutilation showing nail bed damage on multiple digits. Anti-inflammatory carprofen treatment usually alleviated this behavior. (**C**,**D**) Severe self-mutilation. Rats like that shown in (**C**) did not respond well to carprofen treatment and typically had swollen digits and possible ulceration. Rats like that shown in (**D**) typically self-mutilated till bone exposure and possible digit loss. Rats that progressed to severe conditions shown in (**C**) or (**D**) were euthanized in accordance with IACUC guidelines prior to the intended endpoint. Image (**D**) is intentionally blurred according to IACUC guidelines. Scale bar = 5 mm.

Self-mutilation was defined as soft tissue damage typically affecting digits 3–5 of the operated hind paw, ranging in severity as illustrated in Figure 1. Uninjured paws are shown in Figure 1A. Rats showing early signs of self-mutilation (typically nail bed bleeding; Figure 1B) immediately received additional PO carprofen treatment as determined by veterinarian staff. Rats that did not respond well to carprofen and continued to self-mutilate (Figure 1C) or those exhibiting bone exposure (Figure 1D) were humanely euthanized. Minor nail injuries or heel chafing were not classified as self-mutilation. No self-mutilation occurred on unoperated hindlimbs in any rat.

The rate of self-mutilation was defined as the proportion of rats that had any self-mutilation at any time during the study. A rat that showed recurrent self-mutilation behavior was counted as a single subject of that behavior. The onset of self-mutilation behavior was defined as the number of PO days until its first occurrence. The rate of severe self-mutilation was defined as the proportion of rats that progressed to the levels of tissue damage shown in Figure 1C,D.

### 2.6. Statistical Analyses

Behavioral data (SFI and VF) were analyzed using mixed-effects models followed by *post hoc* Tukey’s multiple comparisons test (Prism 8, GraphPad Software, Boston, MA, USA). Behavioral data at specific time points were analyzed using one-way ANOVA followed by Bonferroni’s multiple comparisons tests. Group-level comparisons were assessed using Fisher’s exact tests and Welch’s *t*-tests. Statistical significance was defined as *p* < 0.05. Data are presented as mean ± SEM or as percentages as described in the figure legends. Note that sample sizes in some graphs/tables decrease at later PO times due to early euthanasia, sample harvesting, etc.

Regression analyses were performed using the XLMiner Analysis ToolPak (Frontline Systems Inc., Incline Village, NV, USA) to confirm group-level comparisons and to estimate the independent effects of tested variables on self-mutilation outcomes. Logistic regression models were performed to assess self-mutilation rates and severity, and linear regression model was performed to assess self-mutilation onset. Model assumptions were verified by assessing multicollinearity, model fit, and residual distributions, with no violations detected. Statistical significance was defined as *p* < 0.05. For logistic regression analyses, 95% confidence intervals were reported for odds ratios.

## 3. Results

### 3.1. PEG-Fused Rats That Did Not Self-Mutilate Had Better SFI Scores than PEG-Fused Rats That Self-Mutilated

**Figure 2:**
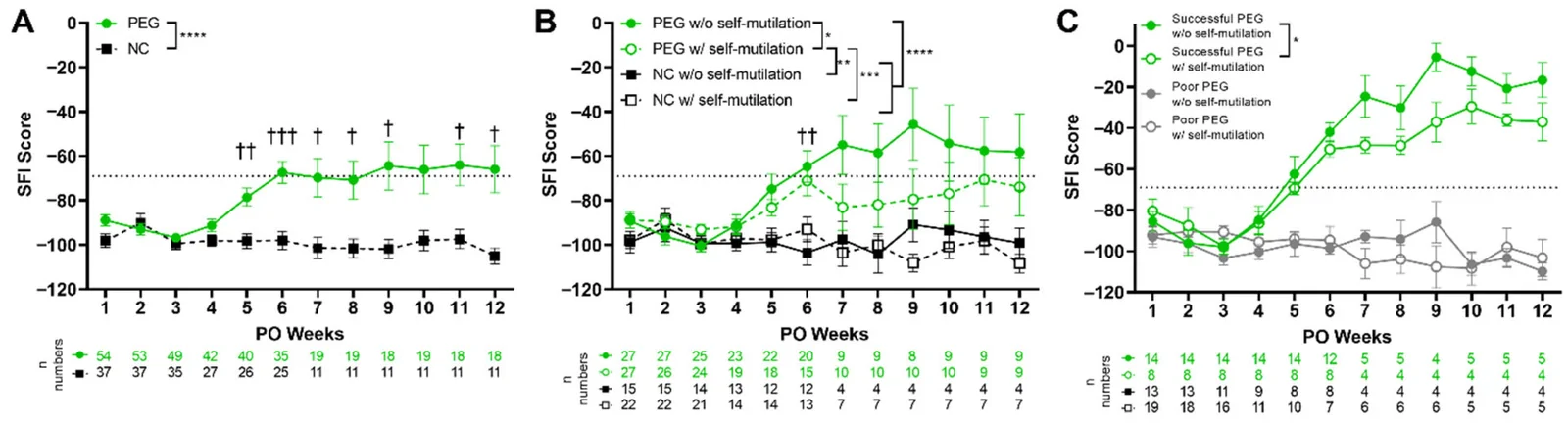
**PEG-fusion repair promotes SFI recovery following sciatic nerve injury, especially in rats without self-mutilation.** In all panels, the dashed line at an SFI score of −69 represents the threshold for successful recovery. (**A**) Weekly SFI comparisons of PEG-fusion (green) vs. Negative Control (NC; black) repairs. NC rats received all PEG-fusion solutions except the solution containing PEG, a membrane fusogen. (**B**) Effects of self-mutilation. SFI scores of PEG and NC rats that had no self-mutilation (filled symbols) or had (open symbols) self-mutilation. (**C**) SFI scores of PEG-fused rats with and without self-mutilation plotted according to whether the animal had successful SFI recovery of −69 (green) or did not recover to −69 (grey). *s indicate significance of the treatment effects (mixed effects model). †s indicate significance at individual time points between PEG and NC groups (Tukey’s multiple comparison). * and †: *p* < 0.05, ** and ††: *p* < 0.01, *** and †††: *p* < 0.001, ****: *p* < 0.0001. Sample sizes (n) are shown below the x-axes. Data given as mean ± SEM. w/o: without; w/: with.

Figure 2 shows SFI recovery of PEG-fused and NC rats (Figure 2A), PEG-fused and NC rats that did or did not have self-mutilation (Figure 2B), and PEG-fused rats with successful or poor SFI scores that did or did not have self-mutilation (Figure 2C). As previously reported [10,11,16,17], PEG-fused rats had significantly better (*p* < 0.0001, mixed-effects model) SFI recovery than NC rats (Figure 2A). We then analyzed SFI recovery in greater detail with respect to self-mutilation (Figure 2B). PEG-fused rats that had no self-mutilation had significantly better (*p* < 0.05, mixed-effects model) SFI recovery than those that had self-mutilation. As expected, self-mutilation had no impact on NC rats, none of which had significant recovery of SFI scores by 12 w PO, similar to previous reports [12,16,17].

We defined long-term successful vs. poor PEG-fusion as maintaining vs. not maintaining an SFI score greater than −69 after 6 w PO [12,16]. Groups of successful and poor PEG-fused rats were analyzed with respect to self-mutilation (Figure 2C). Successful PEG-fused rats that had no self-mutilation had significantly better (*p* < 0.05, mixed-effects model) average SFI scores than those that had self-mutilation. Poor PEG-fused rats that did or did not have self-mutilation, both below the recovery threshold, showed no difference in average SFI scores.

### 3.2. Self-Mutilation Was Associated with Worse VF Recovery in NC Rats but Not in PEG-Fused Rats

**Figure 3:**
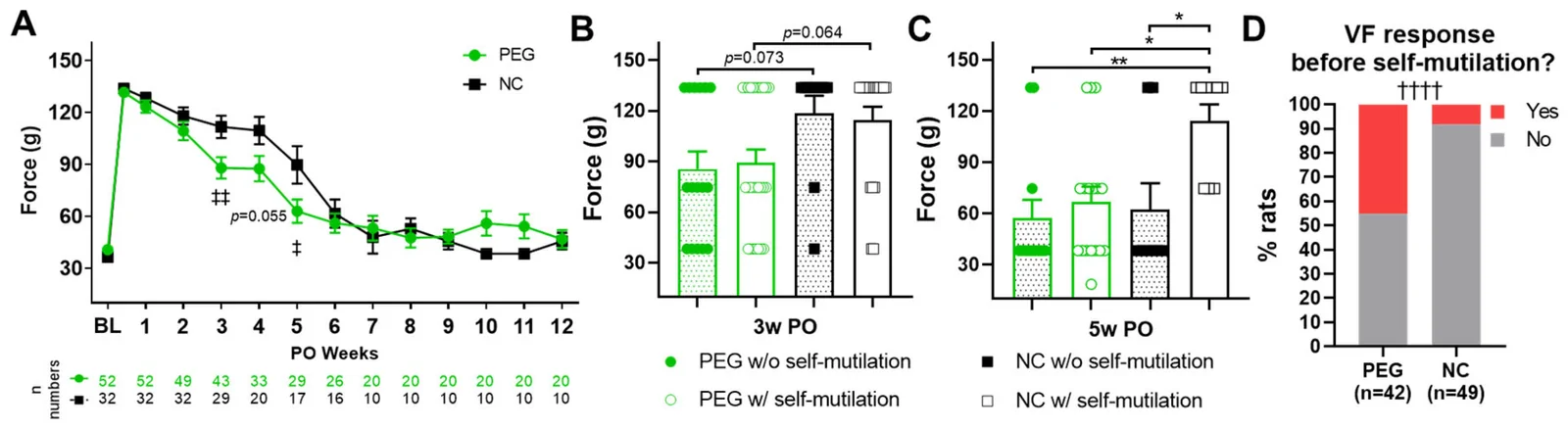
**Self-mutilation is not associated with delayed VF recovery following PEG-fusion but is associated with further delayed VF recovery following NC repairs.** (**A**) Weekly VF responses following PEG-fusion (green) vs. NC (black) repairs. ‡s indicate significance at individual time points (Tukey’s multiple comparison). ‡: *p* < 0.05, ‡‡: *p* < 0.01. Sample sizes (n) are shown below the x-axis. (**B**,**C**) Effects of self-mutilation on VF recovery at (**B**) 3 w PO and (**C**) 5 w PO. Filled symbols: *had no* self-mutilation; open symbols: had self-mutilation. One-way ANOVA. *s indicate Bonferroni’s multiple comparison results; *: *p* < 0.05, **: *p* < 0.01. Data in (**A**–**C**) represent mean ± SEM. (**D**) Sensory recovery before self-mutilation. Percentages of rats within each category are shown. Yes: responded to any tested filament before self-mutilation; No: no response at any time before or the week right after self-mutilation. †s indicate Fisher’s exact test results; ††††: *p* < 0.0001. w/o: without; w/: with.

Figure 3 compares VF recovery of PEG-fused and NC rats (Figure 3A), PEG-fused and NC rats that did or did not have self-mutilation at 3 w PO (Figure 3B) and 5 w PO (Figure 3C). The self-mutilation percentages of PEG-fused and NC rats that regained sensory functions before any self-mutilation were calculated and graphed (Figure 3D). As previously reported [11], PEG-fused rats demonstrated significantly more rapid VF recovery than NC rats (*p* < 0.05, Tukey’s multiple comparisons) before both groups return to baseline VF thresholds (Figure 3A). We also observed no mechanical allodynia, another common symptom of neuropathic pain [56], in either PEG-fused or NC rats.

We now analyzed VF recovery in greater detail with respect to self-mutilation (Figure 3B,C). At 3 w PO (Figure 3B), significant differences in VF threshold among all treatment groups were observed (*p* < 0.05, one-way ANOVA). PEG-fused rats showed marginally significantly lower (*p* > 0.05, Bonferroni’s multiple comparisons) VF threshold than NC rats regardless of self-mutilation. At 5 w PO (Figure 3C), significant differences in VF threshold among all treatment groups were again observed (*p* < 0.01, one-way ANOVA). NC rats that had self-mutilation had a significantly higher (*p* < 0.05, Bonferroni’s multiple comparisons) VF threshold than all other groups. NC rats that had no self-mutilation showed a similar level of VF threshold to PEG-fused groups.

To detect any correlation between VF (sensory) recovery and self-mutilation, we further analyzed VF responses before the occurrence of self-mutilation in rats (Figure 3D). A significantly (*p* < 0.0001, Fisher’s exact test) higher percentage of PEG-fused rats regained some sensory responsiveness than NC rats (45.2% vs. 8.2%, Δ = 37.0%) before self-mutilation occurred. However, VF thresholds between these two groups did not differ significantly (PEG: 57.3 ± 5.0 g; NC: 56.5 ± 10.5 g; *p* > 0.05, Welch’s *t*-test; Figure S1). Together, all these data suggested that sensory recovery assessed by VF testing did not correlate with self-mutilation in PEG-fused rats, but self-militated NC rats had delayed sensory recovery.

### 3.3. PEG-Fusion and VF Testing, but Not Sex or Injury Type, Affect the Rate and Severity of Self-Mutilation in SD Rats with Sciatic ST- or SL-PNIs

We analyzed self-mutilation data from a yet-larger cohort of SD rats (*n* = 325) that included those described in the previous section (*n* = 91). All surgeries were performed by three experienced surgeons. Table 1 summarizes the rates of self-mutilation and severe self-mutilation that required early euthanasia in SD rats across four variables: sex, injury type, repair type, and pre-surgical assignment to a weekly VF testing protocol. We first ran pairwise Fisher’s exact tests as a preliminary analysis, which showed significantly higher self-mutilation rate and severity in NC repairs than PEG-fusion repairs (rate: 69.0% vs. 51.6%, Δ = 17.4%, *p* < 0.01; severity: 32.0% vs. 19.1%, Δ = 12.9%; *p* < 0.05); higher in rats tested for VF than those not tested (rate: 68.1% vs. 50.5%, Δ = 17.6%; *p* < 0.01; severity: 36.1% vs. 15.5%, Δ = 20.6%; *p* < 0.0001); and higher in males than females (rate: 69.7% vs. 52.1%, Δ = 17.6%; *p* < 0.01; severity: 34.8% vs. 18.6%, Δ = 16.2%; *p* < 0.01). No significant difference was observed between rats receiving ST-PNIs and SL-PNIs (rate: 57.1% vs. 56.4%, Δ = −0.7%; *p* > 0.05; severity: 23.2% vs. 22.8%, Δ = 0.4%; *p* > 0.05).

To more completely determine whether each variable independently influenced self-mutilation outcomes and to estimate their effect sizes on self-mutilation outcomes, we further analyzed the dataset in Table 1 using multivariate logistic regression models (Table 2) that are more robust than Fisher’s exact tests. We did not include any of the interaction terms among the variables (sex, injury type, repair method, and VF tests) in Table 2 because none reached statistical significance (*p* > 0.05) or improved the overall fit of these models.

Table 2 shows that PEG-fusion repair was a significant predictor of reduced self-mutilation in rats (*p* < 0.05; OR = 0.524) and was marginally associated with its *non*-progression to severe self-mutilation (*p* = 0.052; OR = 0.564). That is, PEG-fusion repair decreased the odds of self-mutilation by 47.6% and decreased the odds of its progression to severe self-mutilation by 43.6% compared to NC repair. Table 2 also shows that the VF test assignment was marginally associated with the occurrence of self-mutilation (*p* = 0.057; OR = 1.698) and a significant predictor of its progression to severe self-mutilation (*p* < 0.01; OR = 2.638). However, the VF test assignment had the opposite effect of PEG-fusion, producing 69.8% increased odds of self-mutilation and 163.8% increased odds of progression to severe self-mutilation compared to rats that were not assigned VF tests. Injury type (ST-PNI vs. SL-PNI) was not a significant predictor (*p* > 0.05) of self-mutilation or severe self-mutilation. In both logistic regression models, sex was not a significant predictor (*p* > 0.05) of the rates of self-mutilation or severe self-mutilation. Therefore, we conclude that sex had no independent effect, and that the significance observed in the pairwise Fisher’s exact test shown in Table 1 probably reflected the influence of other covariates.

### 3.4. Onset Times of Self-Mutilation in SD Rats Do Not Correlate with Other Variables Tested

The onset of self-mutilation in SD rats ranged from 3 to 43 days PO (Supplementary Table S1), with an average of 18.8 ± 0.7 days. None of the four tested variables significantly (*p* > 0.05) influenced self-mutilation onset based on Fisher’s exact tests (Table S1) or linear regression analyses (Table S2).

### 3.5. Animal Strain and Surgical Experience Correlate with Self-Mutilation Rate and Its Severity

**Figure 4:**
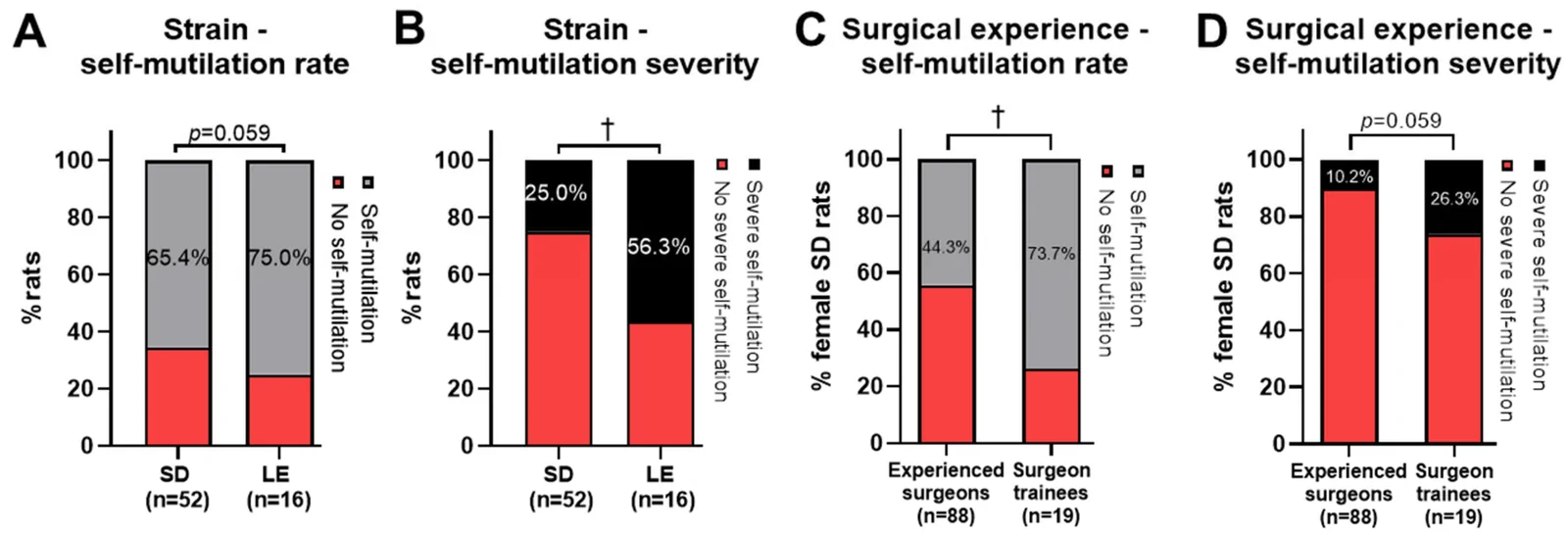
**Strain and surgical experience affect self-mutilation rate and severity.** (**A**,**B**) Strain effect on self-mutilation rate (**A**) and severity (**B**) among NC-repaired Sprague Dawley (SD) and Long Evans (LE) rats without VF tests. SD rats in (**A**,**B**) were a subset of all sciatic-operated rats chosen to match the operation conditions of LE rats. Lewis rats did not self-mutilate (0%) and were not included in (**A**,**B**) for statistical comparisons. (**C**,**D**) Effect of surgical experience on self-mutilation rate (**C**) and severity (**D**) in female SD rats with simple transection PNIs followed by PEG-fusion repair without VF tests. SD rats in (**C**,**D**) were a subset of all sciatic-operated rats chosen to match the operation conditions of rats operated on by surgeon trainees. †s indicate Fisher’s exact test results; †: *p* < 0.05.

We avoided variables that affected self-mutilation (PEG-fusion and VF tests, as described in Table 2) and generated only NC-repaired SD and LE rats without VF tests for strain comparisons (Figure 4A,B). Self-mutilation rates were marginally significantly less for SD rats than for LE rats (65.4% vs. 75.0%; Δ = 9.6%; *p* = 0.059, Fisher’s exact test; Figure 4A). The severe self-mutilation rate was significantly higher for LE rats than for SD rats (56.3% vs. 25.0%, Δ = −31.3%; *p* < 0.05, Fisher’s exact test; Figure 4B). The average onset time for self-mutilation did not differ between SD and LE rats (18.6 ± 1.9 days vs. 17.8 ± 3.9 days; *p* > 0.05, Welch’s *t*-test; Figure S2).

We also examined the rate of self-mutilation with respect to surgical experience in PEG-fusion repair, as assessed by the ability of a surgeon to produce successful SFI recovery after PEG-fusion repairs [15] (Figure 4C,D). Each of the four surgeon trainees performed between two to eight PEG-fusion surgeries following ST-PNIs in female SD rats without VF tests (*n* = 19). These rats were compared with those operated on under identical conditions by the three experienced surgeons described above (*n* = 88). Rats operated on by experienced PEG-fusion surgeons had a significantly lower (44.3% vs. 73.7%, Δ = −29.4%; *p* < 0.05, Fisher’s exact test) self-mutilation rate compared to those operated on by trainees (Figure 4C). Severe self-mutilation rates were marginally significantly less for experienced vs. trainee surgeons (10.2% vs. 26,3%, Δ = −16.1%; *p* = 0.059, Fisher’s exact test; Figure 4D).

## 4. Discussion

We know of no study that has systematically examined the relationship between self-mutilation and sensorimotor recovery following sciatic PNIs. The SFI incorporates both motor control and proprioception of the hind paw and therefore serves as a useful measure of overall sensorimotor functions. In the current study, neurorrhaphy-only NC repair produced poor SFI recovery, with an averaged SFI score remaining about −100 (limb function impaired) at all times after repair, whether or not rats exhibited self-mutilation. This result is consistent with the limited efficacy of neurorrhaphy-only repair reported in both rats and humans [5,6] and suggests that self-mutilation is not associated with SFI recovery in NC rats. In contrast, PEG-fusion repair produced substantial sensorimotor improvement. PEG-fused rats that had no self-mutilation showed significantly better SFI recovery than those that had self-mutilation. However, because some PEG-fused rats that self-mutilated still achieved satisfactory SFI scores, causality between self-mutilation and SFI recovery cannot be established. Additionally, early euthanasia of severely self-mutilated rats prevented assessment of their potential recovery. In summary, the absence of self-mutilation was associated with improved long-term SFI recovery in PEG-fused rats but not in NC rats that showed no SFI recovery whether or not they self-mutilated.

VF filament testing, which assesses mechanical sensitivity and tactile threshold, serves as our second measure of sensory functions. PEG-fusion produced more rapid VF recovery than NC rats. This delayed VF recovery in NC rats was further associated with self-mutilation. Many more NC rats (91.8%) than PEG-fused rats (54.8%) did not regain any level of mechanical sensation prior to the occurrence of self-mutilation. Additionally, none of the mildly self-mutilated rats developed signs of allodynia or hyperalgesia, suggesting that these symptoms of neuropathic pain may have fundamentally different mechanisms than self-mutilation, as previously suggested [42,43,46,57]. In brief, self-mutilation was associated with worse VF sensory recovery in NC rats but not in PEG-fused rats.

A challenge in the field of PEG-fusion research is to consistently translate acute success of PEG-fusion surgeries (confirmed by electrophysiological and axoplasmic reconnection) into long-term sensorimotor recovery. As demonstrated in Figure 2 and Figure 3 and previous studies [10,11,16,17], SFI scores and VF thresholds in successfully PEG-fused rats can range from successful to poor (from +20 to −130 for SFI; from baseline response to no response to any filament for VF). That is, while PEG-fusion, unlike NC repairs, does not limit the capacity or extent of sensorimotor recovery, other unknown factors likely determine whether this potential is realized. Animals experiencing less neuropathic pain may be more willing to engage the affected limb, reinforcing appropriate PNS/CNS synaptic connections that were newly formed following PEG-fusion [10]. Conversely, persistent pain and self-mutilation may limit limb use and impair sensorimotor recovery.

### 4.1. PEG-Fusion Repair Reduces Self-Mutilation Rate and Possibly Severity

Increases in the area and extent of denervation have been reported to increase the rates of self-mutilation [29,55]. Sciatic transections have produced self-mutilation, but saphenous transections have not; injury to both nerves dramatically increases self-mutilation rates, possibly due to increased amount of aberrant firing of sensory neurons associated with a larger area of denervated skin [25]. Because PEG-fusion prevents Wallerian degeneration of many axons and maintains innervation of many distal targets immediately after repair [11,16,17], we hypothesize that PEG-fusion effectively reduces the amount of denervated area and number of aberrant sensory neurons, thereby reducing neuropathic pain and self-mutilation behavior.

### 4.2. Surgical Experience in PEG-Fusion Repair Reduces Self-Mutilation Rate and Possibly Severity

Our hypothesis stated above may also explain why more surgical experience with PEG-fusion correlates with reduced self-mutilation rate and possibly severity. A common technical error among surgeons learning PEG-fusion is misjudging how much epineurium to grasp during suturing when approximating the severed nerve ends. Grasping excessive epineurium compresses the axons and disrupts alignment of the cut axonal surfaces, whereas grasping too little results in inadequate contact between the severed ends. In either case, axons fail to fuse and instead seal following the PEG-fusion step. This error reflects differences between PEG-fusion and conventional repairs with respect to neurorrhaphy. In conventional repairs, surgeons typically grasp much more epineurium to ensure regeneration of proximal axons can occur following distal Wallerian degeneration. Consequently, surgeons experienced in conventional repairs often do not achieve immediate success (restoring electrophysiological and axolemmal continuity) when first performing PEG-fusion repairs. When PEG-fusion fails, rats exhibit outcomes similar to NC repair, i.e., distal axons undergo Wallerian degeneration, distal targets become denervated and atrophy, and little or no SFI recovery occurs [15], which may lead to comparable levels of self-mutilation.

### 4.3. VF Tests Increase Self-Mutilation Severity and Possibly Rate

No previous study has systematically quantified the impact of VF testing on self-mutilation following PNIs [31,32,33,35,36]. In our study, we show that VF tests showed a marginal association with increased self-mutilation rates. VF tests also significantly increased the severity of self-mutilation but did not affect the onset time of self-mutilation. VF tests did not produce self-mutilation in unoperated or sham-operated SD rats, consistent with previous findings [33].

Previous studies hypothesized that increased self-mutilation rates associated with painful stimuli similar to VF tests might be due to peripheral and central sensitization [55,58,59,60]. Stress induced by repeated handling and VF stimulation might also be a contributing factor to self-mutilation because stress exacerbates pain-related behaviors [34] and increases self-mutilation rates in rats following PNIs [33,35,61]. Clinical studies also report that psychological stress can exacerbate neuropathic pain and self-injury behaviors in human patients [44,62,63]. However, our results in another study of Lewis rats suggested that hourly repeated dorsal VF stimulation produced more-rapid desensitization than plantar VF stimulation. Future studies should investigate if the dorsal VF testing protocol produces more stress than plantar VF protocols.

### 4.4. Strain Effects on Self-Mutilation Rate and Severity

In this study, we showed that SD rats possibly have lower rates of mild self-mutilation than LE rats. Significantly more LE rats progressed to severe self-mutilation. In contrast, no Lewis rats had self-mutilation for any condition examined in this study, consistent with previous reports [27,28,29,30,31,32,33,34,35,36]. No self-mutilation occurred following both saphenous and sciatic nerve injuries in Lewis rats (*n* = 20). The same injuries worsened self-mutilation in SD rats (*n* = 3). Genetic analyses suggest a single-gene dominant inheritance produces increased resistance to self-mutilation in Lewis rats [27,28]. Hence, to avoid self-mutilation, Lewis rats may be preferred for studies of long-term recovery after PNIs. However, Lewis rats are capable of self-mutilation when subjected to a yet-more-severe traumatic injury. In an ongoing investigation of the efficacy of PEG-fusion nerve repair (both femoral and sciatic) to successfully transplant autografted hindlimbs, we *have observed self-mutilation of the denervated limb by Lewis rats* (>30%; *n* > 10). That is, the genetic composition of Lewis rats does not *always* protect against self-mutilation.

### 4.5. Injury Type and Sex Do Not Affect Self-Mutilation Rate or Severity

Injury type (ST- vs. SL-PNIs) had no significant effect on self-mutilation, consistent with previous findings that showed no difference in self-mutilation with respect to the length of a gap defect [64]. Previous studies [37,64,65,66] have mixed results on sex effects on self-mutilation. In our study, sex did not independently predict self-mutilation rate or severity in our multivariate regression model (Table 2). These data suggest that the significant association observed in the group-level Fisher’s exact test (Table 1) was probably due to imbalanced sample group sizes and covariation with other factors.

### 4.6. Limitations

This study has several limitations. (1) The exact age of each rat was not recorded as a variable that could affect self-mutilation [29,30]. (2) All injuries were restricted to the sciatic nerve and included only transection and segmental-loss models, but other injury types (e.g., crush or ligation) can also affect self-mutilation [27,28,29,30,31,32,33,34,35,36,67,68]. (3) We used a classification of self-mutilation severity in compliance with current IACUC guidelines that mandate early humane euthanasia instead of the Wall scoring system [29] used in some previous studies [27,28,29,30,31,32,33,34,35,36] that would not meet current IACUC guidelines. Nevertheless, our classification reliably distinguished mild and severe self-mutilation outcomes across six experimental variables. (4) Our regression model did not account for progressive changes in housing conditions (e.g., number of animals per cage and enrichment), analgesic protocols, or inter-operator variations throughout the years following newer IACUC guidelines. (5) Because this retrospective dataset was not originally generated to test the specific hypotheses examined here, the imbalanced group sizes limit the strength of correlation. These results should be confirmed in future, more tightly controlled studies.

### 4.7. Future Directions

Self-mutilation has been observed in human patients following PNIs [41,42,43,44,45,46,47]. Two case reports and two randomized clinical trials have investigated PEG-fusion repair in upper extremity PNIs [49,50,51,52], all reporting significantly earlier sensory recovery following PEG-fusion. While one study found lower Michigan Hand Questionaries pain scores following PEG-fusion [52], none explicitly assessed or documented symptoms related to neuropathic pain. Future PEG-fusion clinical studies should incorporate pain and quality-of-life assessments that are essential to fully characterize the translational safety profile of PEG-fusion. Furthermore, SL-PNIs in the current study were repaired using PEG-fused VPNAs that significantly and consistently reduced self-mutilation across many variables, underscoring both the safety and therapeutic potential of PEG-fused VPNAs.

## Figures and Tables

**Table 1:** **Self-mutilation rates and severity in SD rats across tested variables.**

Independent Variable		Self-Mutilation Rate	Severe Mutilation
**Repair method**	PEG-fusion	51.6% (*n* = 116/225)	19.1% (*n* = 43/225)
	NC	69.0% (*n* = 69/100)	32.0% (*n* = 32/100)
**VF tests**	Assigned	68.1% (*n* = 81/119)	36.1% (*n* = 43/119)
	Not assigned	50.5% (*n* = 104/206)	15.5% (*n* = 32/206)
**Sex**	Female	52.1% (*n* = 123/236)	18.6% (*n* = 44/236)
	Male	69.7% (*n* = 62/89)	34.8% (*n* = 31/89)
**Injury type**	Transection PNI	57.1% (*n* = 128/224)	23.2% (*n* = 52/224)
	Segmental-loss PNI	56.4% (*n* = 57/101)	22.8% (*n* = 23/101)

Table 1 lists the percentages of SD rats that had self-mutilation and severe self-mutilation for a set of independent variables examined in this study. PEG-fusion: polyethylene glycol fusion; NC: Negative Controls; VF: Von Frey; PNI: peripheral nerve injury.

**Table 2:** **Logistic regression analyses of self-mutilation and its progression to severe self-mutilation in SD rats.**

	Self-Mutilation				Severe Self-Mutilation Progression			
	Coefficients (*β*)	Standard Error	OR	95% CI	Coefficients (*β*)	Standard Error	OR	95% CI
**PEG-fusion**	−0.646 *	0.269	0.524	[0.309, 0.888]	−0.573^(*p* = 0.052)^	0.295	0.564	[0.317, 1.004]
**VF tests assigned**	0.530^(*p* = 0.057)^	0.278	1.698	[0.984, 2.929]	0.970 **	0.309	2.638	[1.439, 4.836]
**Transection PNI**	−0.161	0.265	0.851	[0.507, 1.429]	−0.255	0.318	0.775	[0.416, 1.446]
**Female**	−0.370	0.307	0.691	[0.378, 1.261]	−0.292	0.321	0.747	[0.398, 1.402]

*p* values for coefficients are superscripted. * *p* < 0.05, ** *p* < 0.01. Logistic regression statistics: *n* = 325, Chi-Square: left model = 18.4 (*p* < 0.0001); right model = 23.0 (*p* < 0.0001). OR: odds ratio; CI: confidence interval; PEG-fusion: polyethylene glycol fusion; VF: Von Frey; PNI: peripheral nerve injury.

## Data Availability

The data analyzed during the current study are included within the article and Supplementary Material. The raw data supporting the conclusions of this article will be made available by the authors on request.

## Supplementary Materials

The following supporting information can be downloaded at: https://www.mdpi.com/article/10.3390/neurolint18050083/s1, Figure S1: VF thresholds before self-mutilation onset in rats that responded to VF filaments were not different between PEG-fused and NC groups; Figure S2: Average self-mutilation onset did not differ between SD and LE rats; Table S1: Self-mutilation onset in SD rats across tested variables; Table S2: Linear regression analysis of self-mutilation onset in SD rats.

## Abbreviations

The following abbreviations are used in this manuscript:

LE	Long Evans
NC	Negative Control
PEG-fusion or PEG	Polyethylene glycol fusion
PO	Post-operative
SFI	Sciatic Functional Index
SD	Sprague Dawley
SEM	Standard error of the mean
SL-PNI	Segmental-loss peripheral nerve injuries
ST-PNI	Simple transection peripheral nerve injuries
VF	Von Frey
VPNA	Viable peripheral nerve allografts
