# Supplementary material for: PEG-Fusion Repair After Peripheral Nerve Injuries Enhances Behavioral Recovery and Reduces Self-Mutilation in Rat Models
Source: Neurol Int. 2026 Apr 28;18(5):83. doi: 10.3390/neurolint18050083 (PMC13209851; doi:10.3390/neurolint18050083)
Supplement: Supplementary file 1 [file neurolint-18-00083-s001.zip › neurolint-4220040-supplementary.pdf]

Supplementary Materials

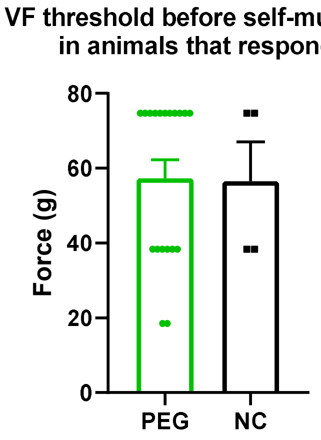

Figure S1. VF thresholds before self-mutilation onset in rats that responded to VF filaments were not different between PEG-fused and NC groups. Welch's t-test. $n = 19$ for PEG-fused group, $n = 4$ for NC group. PEG: polyethylene glycol; NC: Negative Control; VF: Von Frey.

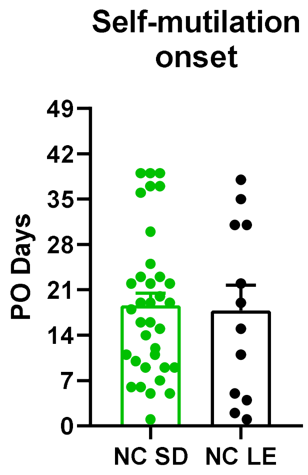

Figure S2. Average self-mutilation onset did not differ between SD and LE rats. Variables that affected self-mutilation (PEG-fusion and VF tests) were avoided, and NC-repaired SD and LE rats without VF tests were compared. Welch's t-test. $n = 35$ for SD rats, $n = 12$ for LE rats. PEG: polyethylene glycol; NC: Negative Control; SD: Sprague Dawley; LE: Long Evans; VF: Von Frey.

Table S1. Self-mutilation onset in SD rats across tested variables

Independent Variable		Onset day (mean ± SEM)	p-value
Repair method	PEG-fusion	20.0 ± 10.1	ns
	NC	17.7 ± 10.6	ns
VF tests	Yes	19.8 ± 10.3	ns
	No	18.1 ± 10.4	ns
Sex	Female	19.5 ± 10.6	ns
	Male	18.7 ± 10.0	ns
Injury type	Transection PNI	17.9 ± 9.9	ns
	Segmental-loss PNI	20.2 ± 10.6	ns

Table S2. Linear regression analysis of self-mutilation onset in SD rats

	Self-mutilation onset			
	Coefficients	Standard Error	t Stat	p-value
PEG-fusion	1.045	1.634	0.639	ns
VF tests	-2.140	1.635	-1.309	ns
Transection PNI	2.164	1.710	1.265	ns
Female	1.071	1.728	0.620	ns
